# Lipid management and cardiovascular risk assessment: Physician perspectives from seven countries across five WHORegions — insights from the INTERASPIRE study

**DOI:** 10.1016/j.ajpc.2026.101576

**Published:** 2026-03-23

**Authors:** Chathurangani Menaka Balasooriya, Catriona Jennings, Eanna Kenny, Dirk De Bacquer, Kausik Kumar Ray, John-Paul Corry, Agnieszka Adamska, Kornelia Kotseva, John W. McEvoy, Chris Noone, Sandra Ganly, Juwairia Alali, Wael Al Mahmeed, Nooshin Bazargani, Junbo Ge, Rose Hui-Chin Jong, Diana Hui-Ping Foo, Yong Huo, Paula Luna Bonilla, Nancy Xinrong Ji, Piotr Jankowski, Yong Li, Amam Mbakwem, Lilian Mbau, Okechukwu Samuel Ogah, Elijah N. Ogola, Adalberto Quintero-Baiz, Mahmoud Umar Sani, Miguel A. Urina-Triana, Renata Wolfshaut-Wolak, Ahmad Syadi Mahmood Zuhdi, David Allan Wood, Jaimini Cegla

**Affiliations:** a Lipids and Cardiovascular Risk Service, Hammersmith Hospital, Imperial College Healthcare NHS Trust, London, UK; b Division of Diabetes, Endocrinology and Metabolism, Imperial College London, London, UK; c Department of Cardiology, School of Medicine, University of Galway, Galway, Ireland; d National Institute for Prevention and Cardiovascular Health, Galway, Ireland; e Department of Public Health and Primary Care, Ghent University, Ghent, Belgium; f Croí Heart & Stroke Centre, Galway, Ireland; g Imperial College Healthcare NHS Trust, London, UK; h Rashid Hospital, Dubai Health, Dubai, , UAE; i Heart and Vascular Institute, Cleveland Clinic Abu Dhabi, Abu Dhabi, United Arab Emirates; j Cardiology Department, Dubai Hospital, Dubai Health, Dubai, UAE; k Department of Cardiology, Zhongshan Hospital Fudan University, Shanghai, China; l Clinical Research Centre, Sarawak General Hospital, Sarawak, Malaysia; m Department of Cardiology, Institute of Cardiovascular Disease, Peking University First Hospital, Beijing, China; n Sociedad Colombiana de Cardiología y Cirugía Cardiovascular, Bogotá, Colombia; o Chinese Cardiovascular Association, Suzhou, China; p Department of Internal Medicine and Geriatric Cardiology, Centre of Postgraduate Medical Education, Warsaw, Poland; q Department of Internal Medicine (Cardiology), Fudan University Huashan Hospital, Shanghai, China; r Department of Medicine, College of Medicine, University of Lagos, Lagos, Nigeria; s Kenya Cardiac Society, Nairobi, Kenya; t Department of Medicine, Cardiology Unit, University of Ibadan, and University College Hospital, Ibadan, Oyo State Nigeria; u Department of Clinical Medicine and Therapeutics, University of Nairobi, Nairobi, Kenya; v Faculty of Health Sciences, University Simon Bolivar, Barranquilla, Atlántico, Colombia; w Department of Medicine, Bayero University Kano and Aminu Kano Teaching Hospital, Kano State, Nigeria; x Faculty of Health Sciences, Institute of Nursing and Midwifery, Jagiellonian University Medical College, Krakow, Poland; y Department of Medicine, Cardiology Unit, University Malaya Medical Centre, Kuala Lumpur, Malaysia

**Keywords:** INTERASPIRE, Cardiovascular risk, LDL-Cholesterol, Non-HDL-Cholesterol, Combination therapy

## Abstract

**Aim:**

Lipid management and risk assessment are key to preventing atherosclerotic cardiovascular disease (ASCVD). As part of the INTERASPIRE study, we conducted a sub-study to evaluate physician practices in cardiovascular risk assessment and lipid management.

**Methods:**

A total of 245 physicians, including cardiologists, general physicians, endocrinologists, and lipidologists across seven countries (China, Colombia, Kenya, Malaysia, Nigeria, Poland, United Arab Emirates) completed a structured questionnaire.

**Results:**

Overall, 87 % of physicians reported estimating ASCVD risk, mainly using ASCVD Risk Estimator Plus (48 %) or SCORE/SCORE2 (26 %). ESC/EAS guidelines were followed by 55 % and AHA/ACC by 52 %. Treatment thresholds varied: 68 % initiated LDL-C lowering at ≥3.0 mmol/L (≥116 mg/dL) in low-risk primary prevention, while 60 % targeted <1.4 mmol/L (<55 mg/dL) in coronary artery disease. Non-HDL-C targets were less frequently applied. Statins predominated (atorvastatin 58 %, rosuvastatin 40 %), but access to advanced agents was uneven: intercountry ranges of PCSK9 inhibitors (7–84 %), inclisiran (0–72 %), and bempedoic acid (0–73 %). Triglyceride therapy was usually initiated at >1.7 mmol/L (>150 mg/dL), mainly with fibrates (71 %). Cardiologists and lipidologists pursued lower LDL-C levels, whereas general physicians were more conservative. Guideline use varied regionally, with ESC guidance dominant in Poland, US guidance in China, Kenya, and the UAE, and national guidelines in Malaysia.

**Conclusion:**

Physicians support risk assessment and statin use, yet wide variation exists in lipid thresholds, non-HDL-C assessment, and access to novel therapies. Despite ESC/EAS and AHA/ACC guidelines uptake, LDL-C targets were often above recommendations, and treatment was predominantly monotherapy. Closing practice gaps requires guideline-aligned tools, stepwise LDL-C lowering, and improved access, particularly in low- and middle-income settings.

**Lay summary:**

Managing lipids and assessing risk are key to preventing disease of the heart and blood vessels caused by blockages (ASCVD).

In this study, doctors from seven countries reported wide differences in how they check heart disease risk, set cholesterol goals, and use treatments.

Most rely on statins, but many start therapy later than guidelines recommend and have uneven access to newer cholesterol-lowering medicines.

Clearer guidance, better education, and improved access to therapies are needed to strengthen prevention worldwide.

## 1 Introduction

Atherosclerotic cardiovascular disease (ASCVD) remains the leading cause of morbidity and mortality worldwide, accounting for an estimated 18 million deaths annually [1]. Despite advances in preventive cardiology, significant gaps persist between evidence-based guideline recommendations and routine clinical practice, particularly in the assessment of cardiovascular (CV) risk, initiation of preventive therapies, and achievement of lipid and blood pressure targets [2,3]. Variability across regions, specialities, and healthcare systems further contributes to inconsistent implementation of strategies aimed at reducing the global burden of ASCVD [4].

Guidelines from major societies—including the European Society of Cardiology/European Atherosclerosis Society (ESC/EAS) and the American Heart Association/American College of Cardiology (AHA/ACC)—emphasise systematic CV risk assessment, early initiation of lipid-lowering therapy in high-risk individuals, and comprehensive lifestyle modification [2,3]. However, real-world physician practices often diverge from these recommendations due to differences in access to diagnostics and therapies, reliance on national guidance, and local resource constraints. Understanding how physicians approach ASCVD prevention across different WHO regions is, therefore, critical to bridging the gap between evidence and practice.

The main INTERASPIRE study, conducted by the National Institute for Prevention and Cardiovascular Health, University of Galway between 2020 and 2023 in partnership with the World Heart Federation, was designed to evaluate secondary prevention of coronary artery disease (CAD) in 14 countries across six World Health Organization (WHO) regions [5]. As part of this study, a sub-study was conducted to examine physicians’ and patients’ perspectives on Lp(a) and ASCVD prevention in selected countries from Africa, the Americas, the Eastern Mediterranean, Europe, and the Western Pacific. Using a structured questionnaire, the physician sub-study explored physician practices in CV risk assessment, treatment thresholds, target goals for lipids, HbA1c, and blood pressure, as well as approaches to lifestyle modification. By providing insights into current global practice patterns, this sub-study highlights both strengths and areas where further harmonisation and system-level support may be needed to improve cardiovascular disease prevention.

## 2 Methods

### 2.1 Data collection

This sub-study of the main INTERARASPIRE study on secondary prevention of coronary heart disease was carried out by the National Institute for Prevention and Cardiovascular Health, University of Galway. Details of the main INTERASPIRE protocol have been reported previously [5]. In summary, eligible patients were identified retrospectively from hospital records if they had experienced either a first or recurrent acute coronary syndrome (STEMI, NSTEMI, or acute myocardial ischaemia) or had undergone elective coronary revascularisation (CABG or PCI) within the past 6 months to 2 years (up to 3 years in Africa). These patients were then invited to attend a structured, standardised study visit that included an interview about their condition and preventive care, along with a clinical examination. This physician survey was conducted as part of the INTERASPIRE Lp(a) sub-study looking at perceptions of both physicians and patients about Lp(a), its management and ASCVD prevention in general. Physicians’ and patients’ perspectives on Lp(a) and its management have been reported separately [6,7].

This sub-study specifically recruited physicians from seven countries across five WHO regions between April and September 2024**:** Kenya and Nigeria (Africa), Colombia (Americas), the UAE (Eastern Mediterranean), Poland (Europe), and China and Malaysia (Western Pacific). The National Coordinators were asked to recruit a convenience sample of three physician groups: 20 cardiologists, 5 lipid specialists (or endocrinologists in countries without lipid specialists, as they would be responsible for managing lipids) and 5 general physicians from hospitals that participated in the main INTERASPIRE study. In some countries, for example Malaysia, no lipid specialist or endocrinologists were recruited as lipids are managed there by cardiologists. The following number of hospitals per each country were involved: Colombia (*n* = 8), China (*n* = 8), Kenya (*n* = 3), Nigeria (*n* = 9), UAE (*n* = 3), Poland (*n* = 5), and Malaysia (*n* = 4). All hospital achieved the target number of physicians, and some recruited more. Trained research assistants conducted telephone interviews in local languages using a standardised questionnaire designed by an international panel of psychologists, cardiologists, lipid experts and a patient representative with dyslipidaemia. Access to therapies was defined as being prescribable via the hospital formulary for outpatients or inpatients. To ensure accuracy, all questionnaires were translated and back-translated, with consistency checks performed by the study nurse coordinator against the original English version.

The sub-study was conducted in accordance with national regulations and applicable ethical requirements in each participating country.

### 2.2 Data management and statistical analyses

Data were collected electronically using a customised platform developed by ARO Specialist Data Centre Services (previously AIMES), Liverpool, UK. All information was reviewed for completeness, internal consistency, and accuracy and stored in compliance with national data protection regulations. Data were summarised as frequencies and proportions. All analyses were carried out at the Statistical Centre, Department of Public Health and Primary Care, Ghent University, Belgium.

To facilitate interpretation of reported LDL-C thresholds and treatment targets, benchmark values from the 2019 ESC/EAS and 2025 ESC/EAS guidelines and 2018 AHA/ACC cholesterol guidelines were prespecified and summarised as reference comparators for primary and secondary prevention (Table 1).

**Table 1:** ESC/EAS and AHA/ACC guideline recommendations for cardiovascular risk assessment and LDL-C management for benchmarking against responses.

	ESC/EAS GUIDELINE	AHA/ACC GUIDELINE
**1. Risk categories**		
	10‑year SCORE2 / SCORE2‑OP risk	10‑year ASCVD risk
	<50 years: <2.5%, 2.5–7.5%, ≥7.5%	High: ≥20%
	50–69 years: <5%, 5–10%, ≥10%	Intermediate: ≥7.5–<20%
	≥70 years: <7.5%, 7.5–15%, ≥15%	Borderline: 5–<7.5%
	(Corresponding to low–moderate, high, and very high risk)	Low: <5%
**2. LDL-C goals and threshold**		
**Primary prevention**	Despite maximally tolerated statin dosage, ≥50% LDL‑C reduction from baseline and LDL‑C goal of: <1.4 mmol/L (55 mg/dL) in very high‑risk groups <1.8 mmol/L (<70 mg/dL) in high‑risk groups <2.6 mmol/L (<100 mg/dL) in moderate‑risk groups <3.0 mmol/L (<116 mg/dL) in low‑risk groups If the goal is not achieved, treatment intensification with non‑statin agents is recommended.	In adults without ASCVD or diabetes with LDL‑C 1.8–4.9 mmol/L (70–190 mg/dL), if patient has ≥20% risk, and In adults with diabetes without ASCVD and LDL‑*C* < 4.9 mmol/L (<190 mg/dL), if ≥50% reduction in LDL‑C or LDL‑*C* < 1.8mmol/L (<70 mg/dL) or non‑HDL‑*C* < 2.6mmol/L (<100 mg/dL) are not achieved despite statin therapy, ezetimibe addition may be reasonable. In adults without ASCVD and LDL‑*C* < 4.9mmol/L (≥190 mg/dL), if ≥50% reduction in LDL‑C or LDL‑*C* < 2.6mmol/L (<100 mg/dL) or non‑HDL‑*C* < 3.4 mmol/L (<130 mg/dL) are not achieved despite statin therapy, non‑statin agents are recommended.
**Secondary prevention**	If LDL‑*C* < 1.4 mmol/L (≥55 mg/dL) despite maximally tolerated statin dosage, addition of ezetimibe or PCSK9 inhibitors after ezetimibe initiation is recommended.	Patients with ASCVD and very high risk: if ≥50% LDL‑C reduction or LDL-*C* < 1.4 mmol/L (<55 mg/dL) are not achieved despite statin therapy, non‑statin agents are recommended.

## 3 Results

### 3.1 Primary prevention (patients with no history of cardiovascular disease): how do clinicians stratify cardiovascular risk and which tools do they use?

Physicians reported diverse guideline use for cardiovascular prevention and lipid management, with most following international recommendations. ESC/EAS (56 %) and AHA/ACC (52 %) were the most cited for cardiovascular prevention, while national guidelines had limited uptake (18 %). A similar pattern was observed in lipid management, with 58% using ESC/EAS and 41 % AHA/ACC. Notably, a small subset of respondents reported not using any guidelines, particularly in lipid care (4 %) (Table 2A).

Speciality influenced guideline choice. Cardiologists favoured ESC/EAS (64 %), general physicians preferred AHA/ACC (58.0 %), and lipidologists used both equally (Table 2A).

Geographic trends were striking; China (88 %) and Kenya (87 %) leaned heavily on AHA/ACC, while Poland naturally preferred ESC/EAS (84 %). Malaysia stood out for its reliance on national guidelines (43 %), suggesting stronger local policy influence (Table 3).

**Table 3:** Country specific differences.

	China	Colombia	Kenya	Malaysia	Nigeria	Poland	UAE
**Cardiovascular prevention guidelines use**							
USA (AHA/ACC/other US guideline)	87.5% (28/32)	59.4% (19/32)	86.7% (26/30)	13.3% (4/30)	52.8% (28/53)	6.3% (2/32)	58.3% (21/36)
ESC Guidelines on CVD Prevention	81.3% (26/32)	59.4% (19/32)	26.7% (8/30)	56.7% (17/30)	35.8% (19/53)	84.4% (27/32)	55.6% (20/36)
Your national guideline	75.0% (24/32)	0.0% (0/32)	6.7% (2/30)	43.3% (13/30)	1.9% (1/53)	12.5% (4/32)	5.6% (2/36)
No guideline	0.0% (0/32)	0.0% (0/32)	0.0% (0/30)	0.0% (0/30)	9.4% (5/53)	0.0% (0/32)	0.0% (0/36)
Other	0.0% (0/32)	0.0% (0/32)	0.0% (0/30)	0.0% (0/30)	3.8% (2/53)	0.0% (0/32)	0.0% (0/36)
**CV risk score**							
WHO Cardiovascular Risk Charts	48.3% (14/29)	3.6% (1/28)	0.0% (0/26)	0.0% (0/26)	10.5% (4/38)	3.2% (1/31)	5.6% (2/36)
SCORE or SCORE 2 (incl SCORE-OP)	37.9% (11/29)	14.3% (4/28)	7.7% (2/26)	3.8% (1/26)	5.3% (2/38)	96.8% (30/31)	19.4% (7/36)
ASCVD Risk Estimator Plus	65.5% (19/29)	64.3% (18/28)	88.5% (23/26)	23.1% (6/26)	50.0% (19/38)	0.0% (0/31)	52.8% (19/36)
Framingham Risk Score	27.6% (8/29)	17.9% (5/28)	3.8% (1/26)	73.1% (19/26)	26.3% (10/38)	0.0% (0/31)	19.4% (7/36)
Reynolds Risk Score	10.3% (3/29)	0.0% (0/28)	0.0% (0/26)	0.0% (0/26)	0.0% (0/38)	0.0% (0/31)	0.0% (0/36)
Globorisk	0.0% (0/29)	0.0% (0/28)	0.0% (0/26)	0.0% (0/26)	0.0% (0/38)	0.0% (0/31)	0.0% (0/36)
Other	3.4% (1/29)	0.0% (0/28)	0.0% (0/26)	0.0% (0/26)	7.9% (3/38)	0.0% (0/31)	11.1% (4/36)
**In a patient with a diagnosis of coronary artery disease what is the recommended target LDL-cholesterol?**							
< 1.0 mmol/L (39 mg/dL)	3.1% (1/32)	3.1% (1/32)	3.3% (1/30)	0.0% (0/30)	5.7% (3/53)	0.0% (0/32)	5.6% (2/36)
< 1.4 mmol/L (55 mg/dL)	50.0% (16/32)	71.9% (23/32)	86.7% (26/30)	80.0% (24/30)	13.2% (7/53)	84.4% (27/32)	63.9% (23/36)
< 1.8 mmol/L (70 mg/dL)	43.8% (14/32)	18.8% (6/32)	10.0% (3/30)	13.3% (4/30)	45.3% (24/53)	15.6% (5/32)	22.2% (8/36)
< 2.6 mmol/L (100 mg/dL)	3.1% (1/32)	3.1% (1/32)	0.0% (0/30)	3.3% (1/30)	28.3% (15/53)	0.0% (0/32)	8.3% (3/36)
< 0.65 mmol/L (25 mg/dL)	0.0% (0/32)	3.1% (1/32)	0.0% (0/30)	0.0% (0/30)	0.0% (0/53)	0.0% (0/32)	0.0% (0/36)
Unsure/don’t know	0.0% (0/32)	0.0% (0/32)	0.0% (0/30)	3.3% (1/30)	7.5% (4/53)	0.0% (0/32)	0.0% (0/36)
**When treating LDL-cholesterol what is your first choice of statin in patients with coronary artery disease?**							
Atorvastatin	65.6% (21/32)	18.8% (6/32)	93.3% (28/30)	93.3% (28/30)	39.6% (21/53)	37.5% (12/32)	75.0% (27/36)
Pitavastatin	3.1% (1/32)	0.0% (0/32)	0.0% (0/30)	0.0% (0/30)	0.0% (0/53)	0.0% (0/32)	0.0% (0/36)
Pravastatin	0.0% (0/32)	0.0% (0/32)	0.0% (0/30)	0.0% (0/30)	0.0% (0/53)	0.0% (0/32)	0.0% (0/36)
Rosuvastatin	25.0% (8/32)	81.3% (26/32)	6.7% (2/30)	6.7% (2/30)	60.4% (32/53)	62.5% (20/32)	25.0% (9/36)
Simvastatin	3.1% (1/32)	0.0% (0/32)	0.0% (0/30)	0.0% (0/30)	0.0% (0/53)	0.0% (0/32)	0.0% (0/36)
Lovastatin	0.0% (0/32)	0.0% (0/32)	0.0% (0/30)	0.0% (0/30)	0.0% (0/53)	0.0% (0/32)	0.0% (0/36)
Fluvastatin	3.1% (1/32)	0.0% (0/32)	0.0% (0/30)	0.0% (0/30)	0.0% (0/53)	0.0% (0/32)	0.0% (0/36)

Most physicians (87 %) routinely assessed CV risk in primary prevention. ASCVD was the most used tool (49 %), followed by SCORE/SCORE2 (27 %) and Framingham (23 %).

Cardiologists led in reporting they used risk scores (90 %), ahead of GPs (84 %) and lipid specialists (83 %). Regionally, Poland favoured SCORE (97 %), Kenya, China, Colombia, UAE, and Nigeria used ASCVD Risk Estimator Plus, and Malaysia favoured Framingham (73 %) (Tables 2A, 3).

**Table 2A:** Physician beliefs on cardiovascular guidelines and risk estimation tools.

Which cardiovascular prevention guidelines do you use?	Cardiologist	General physician	Lipid specialist	Overall
USA (AHA or ACC or other US guideline)	50.4% (68/135)	58.0% (40/69)	48.8% (20/41)	52.2% (128/245)
Joint European Societies (ESC) Guidelines on Cardiovascular Disease Prevention	63.7% (86/135)	43.5% (30/69)	48.8% (20/41)	55.5% (136/245)
Your national guideline	20.0% (27/135)	17.4% (12/69)	17.1% (7/41)	18.1% (46/245)
No guideline	1.5% (2/135)	1.4% (1/69)	4.9% (2/41)	2.0% (5/245)
Other	0.7% (1/135)	0.0% (0/69)	2.4% (1/41)	0.8% (2/245)
**Which lipid guidelines do you use?**				
USA (AHA or ACC or other US guidelines)	37.8% (51/135)	44.9% (31/69)	46.3% (19/41)	41.2% (101/245)
ESC/EAS Guidelines for the Management of Dyslipidaemias	68.1% (92/135)	43.5% (30/69)	51.2% (21/41)	58.4% (143/245)
Your National guideline	19.3% (26/135)	17.4% (12/69)	17.1% (7/41)	18.4% (45/245)
Other	0.0% (0/135)	1.4% (1/69)	4.9% (2/41)	1.2% (3/245)
No guideline	2.2% (3/135)	4.3% (3/69)	7.3% (3/41)	3.7% (9/245)
**In a patient with no history of cardiovascular disease do you estimate total cardiovascular risk as a guide to treatment in primary prevention of CVD?**				
Yes	90.4% (122/135)	84.1% (58/69)	82.9% (34/41)	87.3% (214/245)
If yes, which cardiovascular risk score do you use?				
WHO Cardiovascular Risk Charts	12.3% (15/122)	8.6% (5/58)	5.9% (2/34)	10.3% (22/214)
SCORE or SCORE 2 (including SCORE-OP)	32.8% (40/122)	13.8% (8/58)	23.5% (8/34)	26.6% (57/214)
ASCVD Risk Estimator Plus	48.4% (59/122)	51.7% (30/58)	44.1% (15/34)	48.6% (104/214)
Framingham Risk Score	22.1% (27/122)	31.0% (18/58)	14.7% (5/34)	23.4% (50/214)
Reynolds Risk Score	1.6% (2/122)	1.7% (1/58)	0.0% (0/34)	1.4% (3/214)
Globorisk	0.0% (0/122)	0.0% (0/58)	0.0% (0/34)	0.0% (0/214)
Other	2.5% (3/122)	3.4% (2/58)	8.8% (3/34)	3.7% (8/214)
If yes, what level of total cardiovascular risk would you consider high enough in a middle aged (50- 69 years) person to justify intervention beyond lifestyle with evidence based cardioprotective drug therapies?				
2.5%	4.9% (6/122)	0.0% (0/58)	0.0% (0/34)	2.8% (6/214)
5%	20.5% (25/122)	13.8% (8/58)	35.3% (12/34)	21.0% (45/214)
7.5%	31.1% (38/122)	34.5% (20/58)	38.2% (13/34)	33.25 (71/214)
10%	34.4% (42/122)	32.8% (19/58)	17.6% (6/34)	31.3% (67/214)
15%	4.9% (6/122)	8.6% (5/58)	0.0% (0/34)	5.1% (11/214)
Other	2.5% (3/122)	5.2% (3/58)	5.9% (2/34)	3.7% (8/214)
Unsure/don’t know	1.6% (2/122)	5.2% (3/58)	2.9% (1/34)	2.8% (6/214)

Lipid testing was nearly universal: 96 % requested HDL-C and 91 % ordered triglycerides.

### 3.2 Primary prevention (patients with no history of cardiovascular disease: when do clinicians start therapy and what targets do they aim for?

#### 3.2.1 Risk thresholds for commencing therapy

Treatment thresholds varied: 31 % intervened at ≥10 % risk, 33 % at 7.5 %, and lipidologists were more proactive, with 35 % treating at 5 % or more 10-year risk for ASCVD. (Table 2A).

#### 3.2.2 Lifestyle priorities

Most physicians rated tobacco abstinence**,** a healthy diet low in saturated fat**,** regular physical activity**,** and maintenance of a healthy weight as very important***.***

#### 3.2.3 Lipid management thresholds

**Fig. 1:**
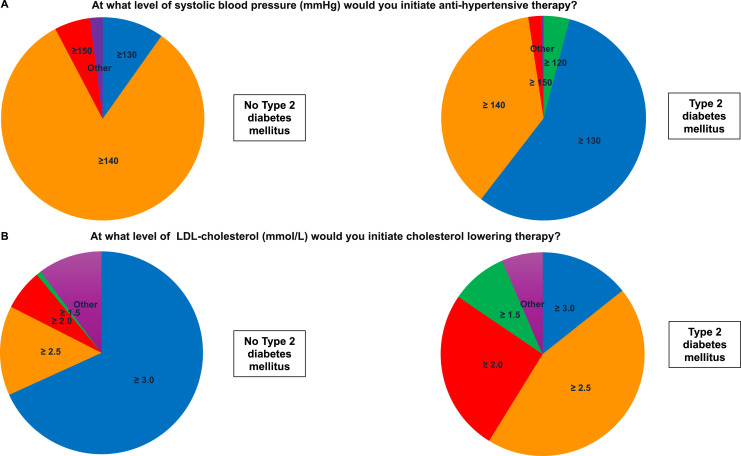
Physician management of patients without coronary artery disease: A: Systolic blood pressure, B: LDL-Cholesterol.

LDL-C therapy began at ≥3.0 mmol/L (≥116 mg/dL) in 68% of non-diabetic patients and at ≥2.5 mmol/L (≥97 mg/dL) in 45 % of those with type 2 diabetes (Fig. 1). Among specialties, 65 % of cardiologists, 75 % of general physicians, and 66 % of lipidologists reported initiating therapy at ≥3.0 mmol/L (≥116 mg/dL) in patients without diabetes. In patients with type 2 diabetes, 45 % of cardiologists, 41 % of general physicians, and 49 % of lipidologists reported initiating therapy at ≥2.5 mmol/L (≥97 mg/dL). Across countries, more than half of physicians reported initiating therapy at ≥3.0 mmol/L (≥116 mg/dL) in patients without diabetes. However, in patients with type 2 diabetes, thresholds varied, with most physicians selecting cut-offs of either ≥2.0 mmol/L (≥77 mg/dL) or ≥2.5 mmol/L (≥97 mg/dL) (Tables 2B, 3).

**Table 2B:** Physician beliefs on LDL-C treatment thresholds and drug management strategies.

In a patient with a diagnosis of coronary artery disease what is the recommended target LDL-cholesterol?		Cardiologist	General physician	Lipid specialist	Overall
< 1.0 mmol/L (39 mg/dL)	4.4% (6/135)		1.4% (1/69)	2.4% (1/41)	3.3% (8/245)
< 1.4 mmol/L (55 mg/dL)	66.7% (90/135)		50.7% (35/69)	51.2% (21/41)	59.6% (146/245)
< 1.8 mmol/L (70 mg/dL)	24.4% (33/135)		23.2% (16/69)	36.6% (15/41)	26.1% (64/245)
< 2.6 mmol/L (100 mg/dL)	4.4% (6/135)		15.9% (11/69)	9.8% (4/41)	8.6% (21/245)
Other	0.0% (0/135)		1.4% (1/69)	0.0% (0/41)	0.4% (1/245)
Unsure/don’t know	0.0% (0/135)		7.2% (5/69)	0.0% (0/41)	2.0% (5/245)
**When treating LDL-cholesterol what is your first choice of statin in CAD patients?**					
Atorvastatin	57.8% (78/135)		69.6% (48/69)	41.5% (17/41)	58.4% (143/245)
Pitavastatin	0.7% (1/135)		0.0% (0/69)	0.0% (0/41)	0.4% (1/245)
Pravastatin	0.0% (0/135)		0.0% (0/69)	0.0% (0/41)	0.0% (0/245)
Rosuvastatin	40.7% (55/135)		30.4% (21/69)	56.1% (23/41)	40.4% (99/245)
Simvastatin	0.7% (1/135)		0.0% (0/69)	0.0% (0/41)	0.4% (1/245)
Lovastatin	0.0% (0/135)		0.0% (0/69)	0.0% (0/41)	0.0% (0/245)
Fluvastatin	0.0% (0/135)		0.0% (0/69)	2.4% (1/41)	0.4% (1/245)
**What other LDL-cholesterol lowering drugs are you able to prescribe from your hospital formulary?**					
Inclisiran	39.3% (53/135)		8.7% (6/69)	29.3% (12/41)	29.0% (71/245)
Proprotein convertase subtilisin/kexin type 9 (PCSK9) inhibitors	60.0% (81/135)		33.8% (23/68)	48.8% (20/41)	50.8% (124/245)
Bempedoic acid	11.1% (15/135)		21.7% (15/69)	7.3% (3/41)	13.5% (33/245)
Fibrates	75.6% (102/135)		71.0% (49/69)	70.7% (29/41)	73.5% (180/245)
Omega-3 fatty acids	52.6% (71/135)		39.1% (27/69)	61.0% (25/41)	50.2% (123/245)
Cholesterol absorption inhibitors	66.7% (90/135)		46.4% (32/69)	51.2% (21/41)	58.4% (143/245)
Bile acid sequestrants	36.3% (49/135)		43.5% (30/69)	22.0% (9/41)	35.9% (88/245)
Resins	8.1% (11/135)		7.2% (5/69)	4.9% (2/41)	7.3% (18/245)
Nicotinic acid	20.7% (28/135)		17.4% (12/69)	7.3% (3/41)	17.6% (43/245)
**What non-HDL-C target do you try and achieve in your patients with coronary artery disease?**					
< 2.2 mmol/L (85 mg/dL)	49.6% (67/135)		36.2% (25/69)	48.8% (20/41)	45.7% (112/245)
< 2.6 mmol/L (100 mg/dL)	17.8% (24/135)		23.2% (16/69)	19.5% (8/41)	19.6% (48/245)
< 3.4 mmol/L (131 mg/dL)	8.9% (12/135)		15.9% (11/69)	0.0% (0/41)	9.4% (23/245)
No target	22.2% (30/135)		23.2% (16/69)	31.7% (13/41)	24.1% (59/245)
Other	1.5% (2/135)		1.4% (1/69)	0.0% (0/41)	1.2% (3/245)
**Would you prescribe a drug to raise HDL-cholesterol levels?**					
Yes	17.8% (24/135)		26.1% (18/69)	29.3% (12/41)	22.0% (54/245)
If yes, which drug class, or drug, would you prescribe?					
Niacin/nicotinic acid	26.1% (6/23)		16.7% (3/18)	8.3% (1/12)	18.9% (10/53)
Statins	56.5% (13/23)		50.0% (9/18)	58.3% (7/12)	54.7% (29/53)
Fibrates	13.0% (3/23)		11.1% (2/18)	33.3% (4/12)	17.0% (9/53)
Evolocumab/Alirocumab	4.3% (1/23)		0.0% (0/18)	0.0% (0/12)	1.9% (1/53)
Other	0.0% (0/23)		22.2% (4/18)	0.0% (0/12)	7.5% (4/53)

However, threshold definitions varied—most considered HDL-*C* < 1.3 mmol/L (<50 mg/dL) in women and <1.0 mmol/L (<40 mg/dL) in men as low. Triglyceride cut-offs also varied, with 78 % using >1.7 mmol/L (>150 mg/dL) and 16 % >2.0 mmol/L (>177 mg/dL), while 2 % were unsure.

Physicians generally initiated blood pressure treatment in CAD at ≥140 mmHg (82 %), whereas in patients with diabetes, 56 % initiated treatment at ≥130 mmHg. Blood pressure thresholds were largely consistent across countries and specialties.

### 3.3 Secondary prevention: what treatment targets do clinicians pursue and which therapies do they use?

LDL-C targets in CAD were most commonly <1.4 mmol/L (<55 mg/dL) (60 %) or <1.8 mmol/L (<70 mg/dL) (26 %). While <1.4 mmol/L (<55 mg/dL) was the predominant target across most countries, Nigerian physicians more commonly used *a* < 1.8 mmol/L (<70 mg/dL) threshold, with only 13 % targeting <1.4 mmol/L (<55 mg/dL) (Tables 2B, 3). Across specialties, <1.4 mmol/L (<55 mg/dL) was also the predominant target, selected by 67 % of cardiologists, 51 % of general physicians, and 51 % of lipidologists.

Non-HDL-C thresholds <2.2 mmol/L (<85 mg/dL) were applied by 46 % of respondents, though 24 % reported not using any specific target. Country-level variation was notable: these targets were frequently used in Colombia (78 %) and Malaysia (73 %), whereas a significant proportion in China (41 %) and Nigeria (40 %) reported no defined non-HDL-C goal (Table 2B).

**Fig. 2:**
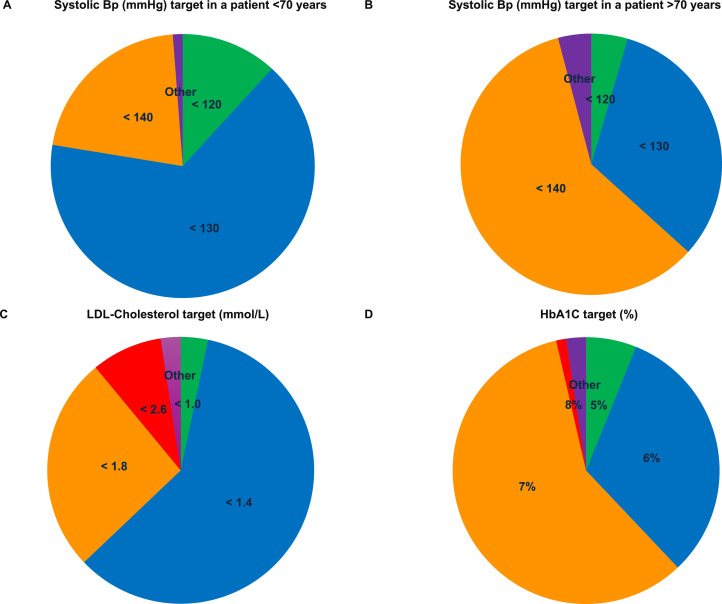
Physician management of patients with coronary artery disease : A, B: Systolic blood pressure, C: LDL-Cholesterol, D: HbA1C.

In patients with CAD, most physicians targeted a systolic blood pressure <130 mmHg in those under 70 years (66 %) and <140 mmHg in those aged ≥70 (60 %). For CAD patients with diabetes, most aimed for an HbA1c <7 % (58 %), while nearly one-third (32 %) pursued a stricter target of <6 % (Fig. 2).

Atorvastatin (58 %) and rosuvastatin (40 %) were the most prescribed statins. General physicians predominantly used atorvastatin (70 %), whereas lipidologists favoured rosuvastatin (56 %). Regionally, atorvastatin use was highest in Kenya and Malaysia (93 %), while rosuvastatin was preferred in Poland, Colombia, and Nigeria. Both statins were commonly used in China and the UAE (Tables 1B,2).

Among non-statin therapies, access varied considerably. Fibrates (74 %) were the most widely available, followed by ezetimibe (58 %), PCSK9 inhibitors (51 %), omega-3 fatty acids (50 %), inclisiran (29 %), and bempedoic acid (13 %). Cardiologists reported the highest access to PCSK9 inhibitors (60 %), compared to lipidologists (49 %) and general physicians (34 %) (Table 2B).

**Fig. 3:**
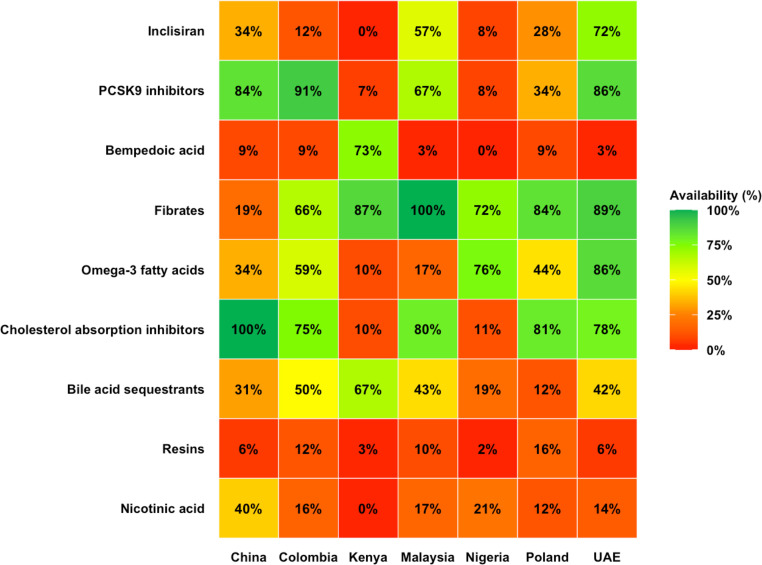
Relative Access to Lipid-Lowering Therapies by country.

Advanced therapies, including PCSK9 inhibitors, were widely available in China, Colombia, and the UAE, but access was more limited in Kenya and Nigeria, where physicians relied primarily on fibrates and omega-3 fatty acids. Although ezetimibe was accessible to over half of respondents, availability remained variable across settings, potentially limiting its broader integration into combination therapy (Table 3, Fig. 3).

### 3.4 Other lipid targets: how are triglycerides and HDL-C managed?

A triglyceride threshold of >1.7 mmol/L (>150 mg/L) was recognized by most physicians (78 %) as indicative of hypertriglyceridemia. Fibrates were the most used therapy (71 %), followed by statins (25 %), icosapent ethyl (12 %), and omacor (5 %). In CAD patients, 71 % of respondents targeted triglyceride levels <1.7 mmol/L (<150 mg/dL). Overall, triglyceride management practices were largely consistent across specialities and countries.

A significant proportion of physicians (22 %) reported treating low HDL-C, with higher rates among lipidologists (29 %). Statins were most frequently used (55 %), followed by niacin (19 %), fibrates (17 %), and PCSK9 inhibitors. The practice appeared to be particularly prominent in Nigeria (64 %), where treatment commonly involved combinations of statins, niacin, and fibrates (Table 1B,2).

## 4 Discussion

This physician survey highlights considerable variation in cardiovascular risk assessment and lipid management practices across healthcare settings. Despite broad awareness of guideline recommendations, differences in the use of risk calculators, LDL-C treatment thresholds, and access to lipid-lowering therapies indicate persistent gaps between evidence and routine clinical practice.

### 4.1 Risk assessment, lifestyle and treatment thresholds in primary prevention

Although most physicians report using formal tools to estimate cardiovascular risk, variation in the choice of risk calculators persists. There remains a divide between the ASCVD Risk Estimator-commonly used in North America and countries outside of Europe—and the SCORE-based models preferred in Europe.

To support greater alignment and enable more consistent application of prevention strategies across European countries, the 2025 ESC guidelines now recommend the use of SCORE2 and SCORE2-OP, which provide calibrated estimates for European populations[2,8].

These findings are consistent with results from earlier studies. In India (2017), physicians showed inconsistent use of risk models and LDL-C targets, with only 59% aligning with guideline-recommended goals [9]. In Kuwait (2015–2016), over 90% reported guideline use, yet Framingham and ACC/AHA tools predominated [10]. In Germany, primary care data showed that just 52% of patients achieved lipid control, with misclassification more frequent in women [11].

This highlights that improving adherence to ASCVD prevention guidelines—whether international or regionally adapted—requires more than dissemination alone. Practical tools, targeted training, and system-level support are essential to bridge the implementation gap globally.

Physicians in this survey strongly endorsed lifestyle modification, consistent with major international prevention guidelines. Both the ESC/EAS and AHA/ACC guidelines emphasise lifestyle intervention-including smoking cessation, healthy diet, weight control, and regular physical activity-as the foundation of cardiovascular disease prevention across all risk categories [2,3]. This alignment suggests that physicians broadly recognise the central role of behavioural strategies in reducing ASCVD risk. Nonetheless, evidence of therapeutic inertia in primary prevention remains evident. In our findings, 68 % of respondents reported initiating lipid-lowering therapy only when LDL-C levels reached ≥3.0 mmol/L (≥116 mg/dL), and 31 % did so only when 10-year cardiovascular risk exceeded 10 % [2]. On the other hand, this may reflect a holistic approach that physicians, not relying on a single number, but assess risk as a whole.

Recent guideline updates aim to address persistent treatment gaps by clarifying thresholds for lipid-lowering therapy and emphasising earlier risk-based intervention. The 2025 ESC focused update defines LDL-C thresholds for pharmacological therapy at ≥1.8 mmol/L(≥70 mg/dL) in very high-risk individuals and ≥2.6 mmol/L (≥100 mg/dL) in high-risk patients [8]. Similarly, the AHA/ACC cholesterol guideline recommends statin therapy based on ASCVD risk assessment and LDL-C thresholds, including consideration of additional therapy when LDL-C remains ≥1.8 mmol/L (70 mg/dL) in very high-risk patients despite maximally tolerated statin therapy [3]. These approaches reflect a shared emphasis on earlier intervention to reduce cumulative exposure to atherogenic lipoproteins and residual cardiovascular risk.

### 4.2 LDL-C targets and combination therapy

Approximately 60 % of physicians targeted LDL-*C* < 1.4 mmol/L (<55 mg/dL) in CAD, consistent with the 2019 ESC/EAS guideline, yet access to combination therapy is severely lacking in some parts of the world. Only 11 % of Nigerians and 10 % of Kenyans had access to the generic drug ezetimibe. Globally, only half of respondents reported access to PCSK9 inhibitors and less than a third to inclisiran. The 2019 guideline recommended adding a PCSK9 inhibitor if LDL-C goals are not met after maximally tolerated statins and ezetimibe [2]. However, the limited availability of these agents may impede goal attainment. Combination lipid-lowering therapy is increasingly emphasised in contemporary guidelines. The 2025 ESC update supports early combination therapy in acute coronary syndrome and introduces additional therapeutic options such as bempedoic acid to facilitate LDL-C goal attainment [8]. Similarly, the AHA/ACC guideline framework recommends intensifying therapy beyond statins when LDL-C levels remain above recommended thresholds, typically through sequential addition of agents such as ezetimibe and PCSK9 inhibitors [3]. Together, these strategies highlight a shift toward more proactive lipid-lowering approaches in high-risk populations.

Still, our findings that 51 % of physicians could prescribe PCSK9 inhibitors and only 14 % could access bempedoic acid suggest that real-world uptake may lag guideline evolution. Variation in practice may also reflect formulary availability and we acknowledge potential structural and reimbursement barriers within different healthcare systems.

### 4.3 Other lipid targets

Non–HDL-C targets were not used by 24 % of physicians, highlighting a potential implementation gap despite ESC/EAS and AHA/ACC guidelines recommending non–HDL-C and ApoB as complementary measures, particularly in hypertriglyceridaemia. This finding may reflect differences in availability of routine lipid measurements, local laboratory reporting practices, lack of familiarity with guideline recommendations, or differences in local practice rather than deliberate deviation from evidence-based guidance.

The majority of respondents recognised triglycerides >1.7 mmol/L (>150 mg/dL) as high and used fibrates, but only 12 % reported using icosapent ethyl. The 2019 ESC/EAS guideline suggested the use of n-3 polyunsaturated fatty acids, particularly icosapent ethyl, in high-risk patients with triglyceride levels between 1.5 and 5.6 mmol/L (135 and 499 mg/dL), and the 2025 ESC update further strengthens this recommendation for high- and very-high-risk individuals [2,8]. Similarly, the AHA/ACC guideline and related scientific statements recognise the role of icosapent ethyl in patients with elevated triglycerides despite statin therapy, reflecting growing evidence supporting triglyceride-targeted risk reduction strategies [3]. Limited access or awareness may explain the low uptake, highlighting an opportunity for education and reimbursement reforms.

Despite the current guideline consensus that low HDL-C is not a primary treatment target, 22 % of surveyed physicians reported treating low HDL-C, with the highest at 29 % among lipid specialists. Statins (55 %) were most used, followed by niacin (19 %) and fibrates. The practice appeared to be particularly prevalent in Nigeria (64 %). Although niacin remains the only agent to significantly raise HDL-C, large-scale trials such as AIM-HIGH and HPS2-THRIVE demonstrated no ASCD benefit, and niacin was subsequently withdrawn from the European market due to safety concerns and lack of efficacy[12]. These findings highlight ongoing confusion around HDL-C management and underscore the need for educational efforts on current evidence and guideline recommendations. However, the reported use of agents such as niacin or fibrates may partly reflect local prescribing environments, differences in guideline implementation, or limited availability of alternative therapies.

### 4.4 Key findings of the survey

This international survey provides insight into physician practices in cardiovascular risk assessment and lipid management across seven countries spanning five WHO regions. Overall, most physicians reported routinely estimating cardiovascular risk and endorsed lifestyle modification as the foundation of cardiovascular disease prevention. However, considerable variation was observed in the choice of risk assessment tools, thresholds for initiating lipid-lowering therapy, and treatment targets for LDL-cholesterol. Access to combination lipid-lowering therapies and newer agents also varied substantially between countries. These findings highlight persistent gaps between guideline recommendations and real-world implementation of lipid management strategies across diverse healthcare settings.

### 4.5 Key implications

The findings of this study suggest that closing the gap between guideline recommendations and clinical practice will require coordinated action at both the clinical and health-system levels. Greater integration of cardiovascular risk assessment tools into electronic health records may facilitate routine and consistent risk estimation during clinical encounters, while structured treatment pathways could support a guideline-aligned, stepwise approach to LDL-C lowering with timely escalation beyond statin therapy when treatment targets are not achieved. In parallel, broader incorporation of non-HDL-C and ApoB measurement may help improve identification and management of residual cardiovascular risk.

Improving access to essential lipid-lowering therapies including ezetimibe, bempedoic acid and PCSK9 inhibitors will also be critical for enabling clinicians to implement guideline-recommended treatment strategies. Educational initiatives, the development of country-specific guidelines aligned with international recommendations, and improved access to diagnostic testing and pharmacological therapies are particularly important in low- and middle-income settings. Together, these measures may help strengthen both primary and secondary prevention strategies and reduce the persistent gap between evidence and routine clinical practice.

### 4.6 Strengths and limitations

Strengths of this survey include its broad geographic scope—spanning seven countries and the inclusion of multiple specialties, offering insight into real-world practice patterns. Comparing results with prior surveys and current guidelines provides a detailed understanding of gaps and progress.

Limitations include modest sample sizes per country and specialty, and due to selection and responder biases the survey may not represent all clinicians and healthcare settings, particularly in rural or under resourced areas. The data were self-reported and therefore subject to reporting bias, and responses may not necessarily reflect actual prescribing behaviour in clinical practice. Differences in interpretation of questionnaire items across languages and healthcare systems may also have influenced responses despite translation and back-translation procedures. In addition, access to lipid-lowering therapies was based on physician report and was not objectively verified against local formulary or reimbursement policies. Finally, the survey design did not assess patient outcomes or longitudinal changes in practice.

## 5 Conclusion

This international survey reveals that physicians universally advocate risk assessment and statin therapy but show substantial variation in LDL-C thresholds, use of non-HDL-C targets, access to combination therapy and adoption of novel agents. Despite a majority reported following the ESC/EAS and AHA/ACC guidelines recommendations, many respondents still initiate therapy at higher LDL-C thresholds and rely on monotherapy. Recent guideline updates, including the 2025 ESC/EAS focused update provides clearer initiation thresholds, endorses early combination therapy in ACS and highlights the use of bempedoic acid and targeted hypertriglyceridemia therapies.

To improve secondary prevention and primary prevention strategies, clinicians and health systems should adopt guideline-aligned risk estimation tools, implement a stepwise approach to LDL-C lowering with timely escalation beyond statin therapy, incorporate non-HDL-C and ApoB measurement, and expand access to novel therapies. Educational initiatives, country-specific guidelines and improved access to diagnostics and drugs particularly in low- and middle-income settings are essential to close the gap between evidence and practice.

## Funding

The INTERASPIRE Lipid Management and Cardiovascular Risk Assessment sub-study was funded by an independent investigator grant from Novartis.

## Data availability

The data underlying this article will be shared on reasonable request to the corresponding author.

**Figure:**
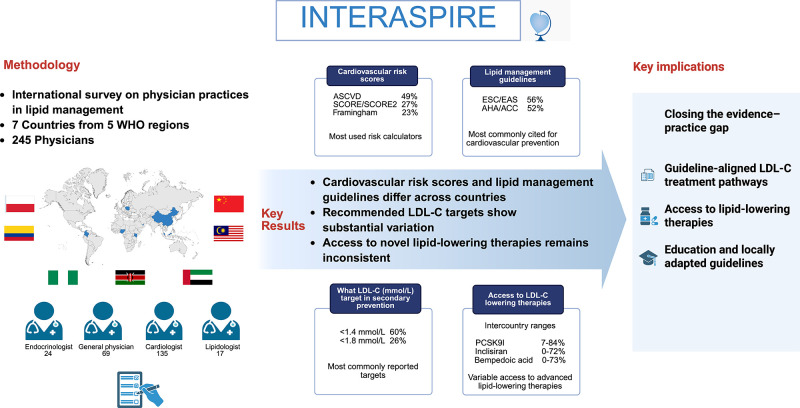
Central illustration.

## CRediT authorship contribution statement

**Chathurangani Menaka Balasooriya:** Writing – original draft, Formal analysis. **Catriona Jennings:** Writing – review & editing, Writing – original draft, Methodology, Formal analysis, Conceptualization. **Eanna Kenny:** Writing – review & editing, Methodology. **Dirk De Bacquer:** Writing – review & editing, Writing – original draft, Methodology, Formal analysis, Conceptualization. **Kausik Kumar Ray:** Writing – review & editing, Writing – original draft, Methodology. **John-Paul Corry:** Writing – review & editing, Methodology. **Agnieszka Adamska:** Writing – review & editing, Methodology. **Kornelia Kotseva:** Writing – review & editing, Methodology. **John W. McEvoy:** Writing – review & editing, Methodology. **Chris Noone:** Writing – review & editing, Methodology. **Sandra Ganly:** Writing – review & editing, Methodology. **Juwairia Alali:** Writing – review & editing, Methodology. **Wael Al Mahmeed:** Writing – review & editing, Methodology. **Nooshin Bazargani:** Writing – review & editing, Methodology. **Junbo Ge:** Writing – review & editing, Methodology. **Rose Hui-Chin Jong:** Writing – review & editing, Methodology. **Diana Hui-Ping Foo:** Writing – review & editing, Methodology. **Yong Huo:** Writing – review & editing, Methodology. **Paula Luna Bonilla:** Writing – review & editing, Methodology. **Nancy Xinrong Ji:** Writing – review & editing, Methodology. **Piotr Jankowski:** Writing – review & editing, Methodology. **Yong Li:** Writing – review & editing, Methodology. **Amam Mbakwem:** Writing – review & editing, Methodology. **Lilian Mbau:** Writing – review & editing, Methodology. **Okechukwu Samuel Ogah:** Writing – review & editing, Methodology. **Elijah N. Ogola:** Writing – review & editing, Methodology. **Adalberto Quintero-Baiz:** Writing – review & editing, Methodology. **Mahmoud Umar Sani:** Writing – review & editing, Methodology. **Miguel A. Urina-Triana:** Writing – review & editing, Methodology. **Renata Wolfshaut-Wolak:** Writing – review & editing, Methodology. **Ahmad Syadi Mahmood Zuhdi:** Writing – review & editing, Methodology. **David Allan Wood:** Writing – review & editing, Writing – original draft, Methodology, Formal analysis, Conceptualization. **Jaimini Cegla:** Writing – review & editing, Writing – original draft, Methodology, Formal analysis, Conceptualization.

## Declaration of competing interest

The authors declare the following financial interests/personal relationships which may be considered as potential competing interests: The INTERASPIRE Lipid Management and Cardiovascular Risk Assessment sub-study reports financial support was provided by Novartis. Kausik Kumar Ray reports a relationship with Amgen,Sanofi,Regeneron,Daiichi Sankyo,Novartis,Kowa,Esperion,Novo Nordisk,MSD, Lilly,Silence,AZ,New Amsterdam,Bayer,Beren, CLEERLY,EMENDOBIO,SCRIBE,CRISPR,VAXXINITY,Amarin,Ultragenix,Cargene, Resverlogix,BI,Viatris,Biologix,Macleod,PEMI31 that includes: consulting or advisory, funding grants, and speaking and lecture fees. Piotr Jankowski reports a relationship with Amgen, Novartis, Servier, and Zentiva that includes: funding grants and speaking and lecture fees. If there are other authors, they declare that they have no known competing financial interests or personal relationships that could have appeared to influence the work reported in this paper.
